# *Streptococcus pluranimalium* 2N12 Exerts an Antagonistic Effect Against the Swine Pathogen *Actinobacillus pleuropneumoniae* by Producing Hydrogen Peroxide

**DOI:** 10.3389/fvets.2021.787241

**Published:** 2021-12-08

**Authors:** Katy Vaillancourt, Michel Frenette, Marcelo Gottschalk, Daniel Grenier

**Affiliations:** ^1^Groupe de Recherche en Écologie Buccale, Faculté de Médecine Dentaire, Université Laval, Quebec City, QC, Canada; ^2^Centre de Recherche en Infectiologie Porcine et Avicole, Fonds de Recherche du Québec–Nature et Technologies, Saint-Hyacinthe, QC, Canada; ^3^Groupe de Recherche sur les Maladies Infectieuses du Porc, Faculté de Médecine Vétérinaire, Université de Montréal, Saint-Hyacinthe, QC, Canada

**Keywords:** *Actinobacillus pleuropneumoniae*, *Streptococcus pluranimalium*, porcine pleuropneumonia, swine infection, hydrogen peroxide, antagonism

## Abstract

*Actinobacillus pleuropneumoniae* is the causal agent of porcine pleuropneumonia, a highly contagious and often deadly respiratory disease that causes major economic losses in the swine industry worldwide. The aim of the present study was to investigate the hydrogen peroxide (H_2_O_2_)-dependent antagonistic activity of *Streptococcus pluranimalium* 2N12 (pig nasal isolate) against *A. pleuropneumoniae*. A fluorimetric assay showed that *S. pluranimalium* produces H_2_O_2_ dose- and time-dependently. The production of H_2_O_2_ increased in the presence of exogenous lactate, suggesting the involvement of lactate oxidase. All 20 strains of *A. pleuropneumoniae* tested, belonging to 18 different serovars, were susceptible to H_2_O_2_, with minimal inhibitory concentrations and minimal bactericidal concentrations ranging from 0.57 to 2.3 mM. H_2_O_2_, as well as a culture supernatant of *S. pluranimalium*, killed planktonic cells of *A. pleuropneumoniae*. Treating the culture supernatant with catalase abolished its bactericidal property. H_2_O_2_ was also active against a pre-formed biofilm-like structure of *A. pleuropneumoniae* albeit to a lesser extent. A checkerboard assay was used to show that there were antibacterial synergistic interactions between H_2_O_2_ and conventional antibiotics, more particularly ceftiofur. Based on our results and within the limitations of this *in vitro* study, the production of H_2_O_2_ by *S. pluranimalium* could be regarded as a potential protective mechanism of the upper respiratory tract against H_2_O_2_-sensitive pathogens such as *A. pleuropneumoniae*.

## Introduction

*Actinobacillus pleuropneumoniae* is a Gram-negative facultative anaerobic encapsulated coccobacillus. It is classified into two biotypes: biotype 1 requires nicotinamide adenine dinucleotide (NAD) to grow while biotype 2 is NAD independent (1). Based on their antigenic composition and the properties of their lipopolysaccharides (LPS) and capsular polysaccharides, nineteen serovars of *A. pleuropneumoniae* have been described to date (2, 3). This bacterium is transmitted from pig to pig mainly by direct oral and nasal contact or by bioaerosols following the introduction of asymptomatic carrier pigs into a herd or piggery (2). *A. pleuropneumoniae* is a respiratory pathogen that colonizes the tonsils and nasal cavities of pigs and causes porcine pleuropneumonia, a highly contagious and often deadly respiratory disease responsible for significant economic losses in the swine industry worldwide (1). This disease, for which the clinical features range from peracute to chronic, is characterized by the presence of hemorrhagic, fibrinous, and necrotic lung lesions. The serovars most often isolated from infections differ depending on the region of the world. In North America, serovars 5 and 7 are predominant while serovars 2 and 9/11 are most often isolated in European countries (2, 4–7). The use of antibiotics at the onset of the disease is the most effective treatment for diminishing the severity of the clinical symptoms, the death rate, and the spread of infections (1, 2).

*A. pleuropneumoniae* produces a broad array of virulence factors that play crucial roles in the infectious process of porcine pleuropneumonia by contributing to colonization, evasion of the immune defense mechanisms, and induction of lung lesions (2, 8, 9). The most important and well-known virulence factors are the Apx toxins (I to III), which belong to the family of Repeats in ToXin (RTX) toxins (9, 10). They cause the lysis of different cell types, including red blood cells, alveolar epithelial cells, neutrophils, and macrophages (9, 10).

A wide variety of bacterial species are present in the upper respiratory tract of pigs (11), and are likely to have positive or negative interactions with *A. pleuropneumoniae*. To the best of our knowledge, the negative interactions, such as competition and antagonism, that occur in the porcine respiratory tract and that involve *A. pleuropneumoniae* have not been thoroughly investigated to date. Antagonism is related to the ability of microorganisms to produce antimicrobial substances, including bacteriocins, organic acids, and hydrogen peroxide (H_2_O_2_) (12, 13). Preliminary analyses in our laboratory showed that *Streptococcus pluranimalium* 2N12, which was isolated from a pig nasal sample, is able to antagonize the growth of *A. pleuropneumoniae* in a deferred growth inhibition assay. In the present study, we investigated the H_2_O_2_-mediated antagonistic activity of *S. pluranimalium* 2N12 against *A. pleuropneumoniae*.

## Materials and Methods

### Bacteria and Growth Conditions

The *A. pleuropneumoniae* strains used in this study (Table 1) were grown in Todd Hewitt Broth (THB; BD-Canada, Mississauga, ON, Canada) supplemented with NAD (20 μg/mL; THB-NAD) and incubated in an anaerobic chamber (80% N_2_, 10% CO_2_, 10% H_2_) at 37°C. *S. pluranimalium* 2N12, which was isolated by our laboratory from the nasal cavity of a healthy pig, was identified by 16S rRNA gene sequencing. It was cultivated in THB and was incubated at 37°C in aerobic conditions.

**Table 1:** Minimum inhibitory concentration (MIC) and minimum bactericidal concentration (MBC) values of H_2_O_2_ against *A. pleuropneumoniae*.

***A. pleuropneumoniae*** **strain**	**H_2_O_2_ (mM)**	
	**MIC**	**MBC**
4074 (serovar 1)	1.15–2.3	1.15–2.3
4226 (serovar 2)	1.15–2.3	1.15–2.3
S1421 (serovar 3)	0.57–1.15	0.57–1.15
M62 (serovar 4)	1.15	1.15
K17 (serovar 5a)	1.15	1.15–2.3
81750 (serovar 5b)	2.3	2.3
L20 (serovar 5b)	1.15	1.15
FEMφ (serovar 6)	0.57	0.57
WF83 (serovar 7)	0.57	0.57
405 (serovar 8)	1.15	1.15
CVJ13261 (serovar 9)	1.15	1.15
D13039 (serovar 10)	1.15–2.3	1.15–2.3
56153 (serovar 11)	1.15	2.3
8328 (serovar 12)	2.3–4.6	2.3–4.6
N-273 (serovar 13)	1.15	1.15
3906 (serovar 14)	0.57–1.15	0.57–1.15
HS143 (serovar 15)	1.15–2.3	1.15–2.3
A85/14 (serovar 16)	0.57–1.15	0.57–1.15
162871 (serovar 17)	0.57–1.15	0.57–1.15
7311555 (serovar 18)	1.15	1.15

### Deferred Growth Inhibition Assay for Detecting the Antagonistic Activity of *S. pluranimalium* Against *A. pleuropneumoniae*

Two-μL aliquots of an overnight broth culture of *S. pluranimalium* 2N12 were applied on the surface of THB agar plates. After a 24-h incubation at 37°C to allow bacterial growth, the plates were overlaid with soft THB-NAD agar (0.75%, w/v) that had been inoculated with an overnight culture of either *A. pleuropneumoniae* K17 (biotype 1, serovar 5a), 81750 (biotype 1, serovar 5b), or L20 (biotype 1, serovar 5b) (700 μL of bacterial culture in 7 mL of soft THB-NAD agar), and were incubated for a further 24 h at 37°C. The inhibitory zones (in mm) were measured from the edge of the *S. pluranimalium* growth to the margin of the inhibition area. The effect of adding catalase (100 units/mL) to the THB agar plates when growing *S. pluranimalium* was evaluated. Assays were performed in triplicate, and the means ± standard deviations (SD) were calculated.

### Determination of H_2_O_2_ Production

The quantification of H_2_O_2_ production by *S. pluranimalium* 2N12 was assessed using the LAPTg medium described by Alvarez et al. (14). LAPTg medium contains 1.5% (w/v) peptone, 1% (w/v) tryptone, 1% (w/v) yeast extract, 1% (w/v) glucose, and 0.1% (v/v) Tween 80. The pH was adjusted to 6.5. Briefly, *S. pluranimalium* was cultivated overnight in LAPTg, harvested by centrifugation, and suspended in the same medium at an optical density at 660 nm (OD_660_) of 0.6 (~3 × 10^9^ colony-forming units [CFU]/mL), 0.4, 0.2, and 0.1. The bacteria were incubated at 37°C with shaking (120 RPM), and samples were taken after 0, 1, and 2 h. In one experiment using an inoculum with an OD_660_ of 0.1, the incubation was extended to 24 h. H_2_O_2_ in the cell-free supernatants was determined using a fluorimetric H_2_O_2_ assay kit (Sigma-Aldrich Canada Co., Oakville, ON, Canada) according to the manufacturer's instructions. Assays were performed in triplicate in three independent experiments, and the means ± SD were calculated.

### Effect of Lactate on H_2_O_2_ Production

The effect of exogenous lactate on H_2_O_2_ production by *S. pluranimalium* 2N12 was assessed. Lactate was added at concentrations ranging from 1 to 25 mM to the LAPTg medium, and the production of H_2_O_2_ was quantified after a 2-h incubation following inoculation (OD_660_ = 0.4) as described above. Assays were performed in triplicate in three independent experiments, and the means ± SD were calculated.

### Determination of Minimum Inhibitory Concentrations and Minimum Bactericidal Concentrations of H_2_O_2_

Minimum inhibitory concentrations (MICs) and minimum bactericidal concentrations (MBCs) of H_2_O_2_ (Sigma-Aldrich Canada Co.) against twenty strains of *A. pleuropneumoniae* (Table 1) belonging to eighteen different serovars were determined as follows. Briefly, 24-h bacterial cultures (*A. pleuropneumoniae*) in THB-NAD were diluted in fresh broth medium to obtain an OD_660_ of 0.2. Equal volumes (100 μL) of bacteria and two-fold serial dilutions of 0.1% H_2_O_2_ (corresponding to 29.5 mM) in THB-NAD were mixed in the wells of a 96-well microplate. Control wells with no bacteria or no H_2_O_2_ were also prepared. The microplates were incubated (without agitation) at 37°C for 24 h in an anaerobic chamber prior to determining bacterial growth by recording the OD_660_ using an xMark Microplate spectrophotometer (Bio-Rad Laboratories Inc., Mississauga, ON, Canada). The MIC value was defined as the lowest concentration of H_2_O_2_ that completely prevented bacterial growth. The MBC values were determined by subculturing 5-μL aliquots from each well that exhibited no visible growth on THB-NAD agar plates, which were incubated for 48 h at 37°C in an anaerobic chamber. The MBC value was defined as the lowest concentration of H_2_O_2_ at which no colonies formed. Assays were performed in triplicate in three independent experiments.

### Killing Assay of Planktonic *A. pleuropneumoniae* by H_2_O_2_ and a Culture Supernatant of *S. pluranimalium*

In a first assay, cells from overnight cultures of *A. pleuropneumoniae* 81750 and K17 were harvested by centrifugation and were suspended in LAPTg medium to obtain an OD_660_ of 0.4. The bacterial suspensions (2 mL) were mixed with 6 mL of H_2_O_2_ (in LAPTg medium) to obtain a final concentration of 2.3 mM. The mixtures were incubated anaerobically for 4 h at 37°C prior to assessing bacterial viability by determining the number of CFUs. In a second assay, *S. pluranimalium* 2N12 was cultivated overnight in THB, harvested by centrifugation, and suspended in LAPTg medium to an OD_660_ of 0.4. The bacterial suspension was incubated for 2 h with shaking at 37°C. The cell-free culture supernatant was then harvested, adjusted to pH 7.0, and filter-sterilized. The amount of H_2_O_2_ in the supernatant was quantified using the fluorimetric assay described above. The *A. pleuropneumoniae* suspensions (2 mL, OD_660_ of 0.4) were mixed with 6 mL of the *S. pluranimalium* culture supernatant. The mixture was anaerobically incubated at 37°C for 4 h, and bacterial viability was assessed by determining the CFUs. The effect of pre-treating the culture supernatant of *S. pluranimalium* with catalase (100 units/mL) for 1 h at room temperature prior to performing the assay was assessed. All the assays were performed in triplicate, and the means ± SD were calculated. Given the above viable count methodology, the minimum detection limit is 10^1^ CFU/mL.

### Killing Assay of a Biofilm-Like Structure of *A. pleuropneumoniae* by H_2_O_2_ and a Culture Supernatant of *S. pluranimalium*

*A. pleuropneumoniae* 81750 and K17 biofilm-like structures were pre-formed overnight (THB-NAD, 37°C, anaerobic chamber) in the wells of a 96-well clear bottom black wall microplate (Greiner Bio-One North America, Monroe, NC, USA). After removing planktonic and loosely-attached bacteria by aspiration, the biofilm-like structures were exposed (4 h, 37°C, anaerobic chamber) to either H_2_O_2_ (0.57, 1.15, 2.3, and 4.6 mM) or a culture supernatant of *S. pluranimalium* 2N12 prepared as described above. Biofilm-like structure viability was assessed using the FilmTracer LIVE/DEAD Biofilm Viability kit (Life Technologies Corporation, Eugene, OR, USA) according to the manufacturer's protocol. Assays were performed in triplicate, and the means ± SD were calculated.

### Effect of H_2_O_2_ on the Activity of Conventional Antibiotics Against *A. pleuropneumoniae*

Interactions between H_2_O_2_ and conventional antibiotics (ceftiofur, penicillin G, tetracycline) used to treat *A. pleuropneumoniae* infections were evaluated using the checkerboard technique (15). H_2_O_2_ (from 4.6 mM) was serially diluted two-fold in THB-NAD (100 μL) along the ordinate of a 96-well microplate, while antibiotics (from 2 μg/mL) were serially diluted two-fold in THB-NAD (100 μL) along the abscissa. A cell suspension of *A. pleuropneumoniae* 81750 prepared in THB-NAD and adjusted to an OD_660_ of 0.2 was used as the inoculum. The microplate wells were inoculated with 100 μL of bacterial suspension, and the microplates were incubated at 37°C for 24 h. Wells with no bacteria or compounds were used as controls. After the incubation period, bacterial growth was assessed visually. The lowest concentration at which no growth occurred was considered the MIC. The fractional inhibitory concentration index (FICI) was calculated as follows: FICI = FIC_A_ + FIC_B_ = (MIC_H2O2_ in combination/MIC_H2O2_ alone) + (MIC_Antibiotic_ in combination/MIC_Antibiotic_ alone). An FICI ≤ 0.5 was considered as having a synergistic effect, an FICI > 0.5 and ≤ 1.0 as having an additive effect, an FICI > 1.0 and ≤ 4.0 as having no effect, and an FICI > 4.0 as having an antagonistic effect (15). Two independent assays were performed.

### Effect of Catalase on the Growth of and Biofilm Formation by *S. pluranimalium*

The effect of catalase (6.25, 12.5, 25, 50, 100, and 200 units/mL) on the growth of and biofilm formation by *S. pluranimalium* 2N12 was assessed in a 96-well microplate. Bacteria were cultivated for 20 h, and growth was monitored by recording the OD_660_ using a Synergy 2 microplate reader (BioTek Instruments, Winooski, VT, USA). Planktonic and poorly-attached bacterial cells were then removed by aspiration with a 26g needle. Biofilms were stained with 100 μL of 0.01% crystal violet for 15 min, washed with distilled water, and dried at 37°C for 2 h. To release biofilm-embedded crystal violet, 100 μL of 75% ethanol was added, and the plate was shaken for 15 min. The biofilm biomass was estimated by recording the absorbance at 550 nm (A_550_) using an xMark Microplate spectrophotometer. Assays were performed in triplicate in three independent experiments, and the means ± SD were calculated.

### Statistical Analysis

Statistical analyses were performed using a one-way ANOVA analysis of variance with a *post-hoc* Bonferroni multiple comparison test (GraphPad Software Inc., San Diego, CA, USA). All results were considered statistically significant at *p* < 0.01.

## Results

The ability of *S. pluranimalium* 2N12 to exert an antagonistic effect on *A. pleuropneumoniae* was first examined using a deferred growth inhibition assay. As reported in Table 2, *A. pleuropneumoniae* 81750 and L20 strains, which belong to serovar 5b, displayed inhibitory zones of 4.0 mm and 6.3 mm, respectively. *A. pleuropneumoniae* K17 (serovar 5a) appeared less sensitive, showing an inhibitory zone of 1.3 mm. The deferred growth inhibition assay was then performed in the presence of catalase to determine whether the inhibition of *A. pleuropneumoniae* may have resulted from the production of H_2_O_2_ by *S. pluranimalium*. Adding catalase (100 units/mL) resulted in a significant reduction or complete abolition of the inhibitory zones (Table 2).

**Table 2:** Inhibitory zones produced by *S. pluranimalium* 2N12 against *A. pleuropneumoniae* in a deferred growth inhibition assay.

***A. pleuropneumoniae*** **strain**	**Inhibitory zones in mm (mean ± SD)**	
	**No catalase**	**With catalase**
K17 (serovar 5a)	1.3 ± 0.6	0
81750 (serovar 5b)	4.0 ± 0	0.5 ± 0
L20 (serovar 5b)	6.3 ± 0.6	0.5 ± 0

**Figure 1:**
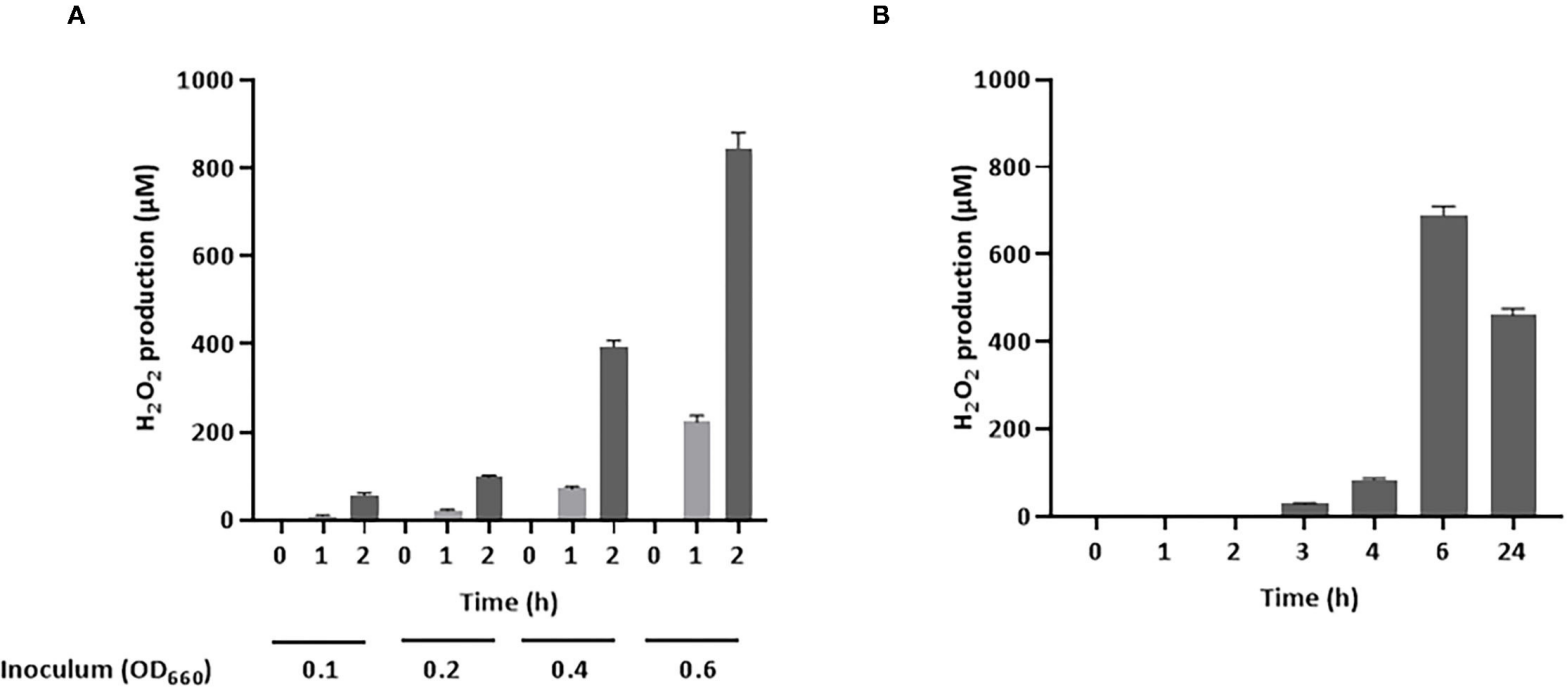
Production of H_2_O_2_ by *S. pluranimalium* 2N12. **(A)**: Dose- and time-dependent effect. **(B)**: Time-dependent effect. H_2_O_2_ was quantified using a fluorimetric assay. Assays were performed in triplicate in two independent experiments, and the means ± SD were calculated.

As H_2_O_2_ is a potential inhibitory compound that is active against *A. pleuropneumoniae*, its production by *S. pluranimalium* 2N12 was confirmed by quantifying H_2_O_2_ levels in a culture supernatant (LAPTg medium) using a fluorimetric assay. As shown in Figure 1A, *S. pluranimalium* dose- and time-dependently produced H_2_O_2_. A low bacterial inoculum (OD_660_ of 0.1) resulted in the production of 56.3 μM of H_2_O_2_ after a 2-h incubation, while the use of a higher bacterial inoculum (OD_660_ of 0.6) was associated with the production of 845.5 μM of H_2_O_2_, a 15-fold increase compared to that of the low bacterial inoculum. It was then determined whether a longer incubation time for the low bacterial inoculum (OD_660_ of 0.1) resulted in the production of higher amounts of H_2_O_2_. The results reported in Figure 1B show that extending the incubation to 24 h was associated with lower amounts of H_2_O_2_ compared to the 6-h incubation time.

**Figure 2:**
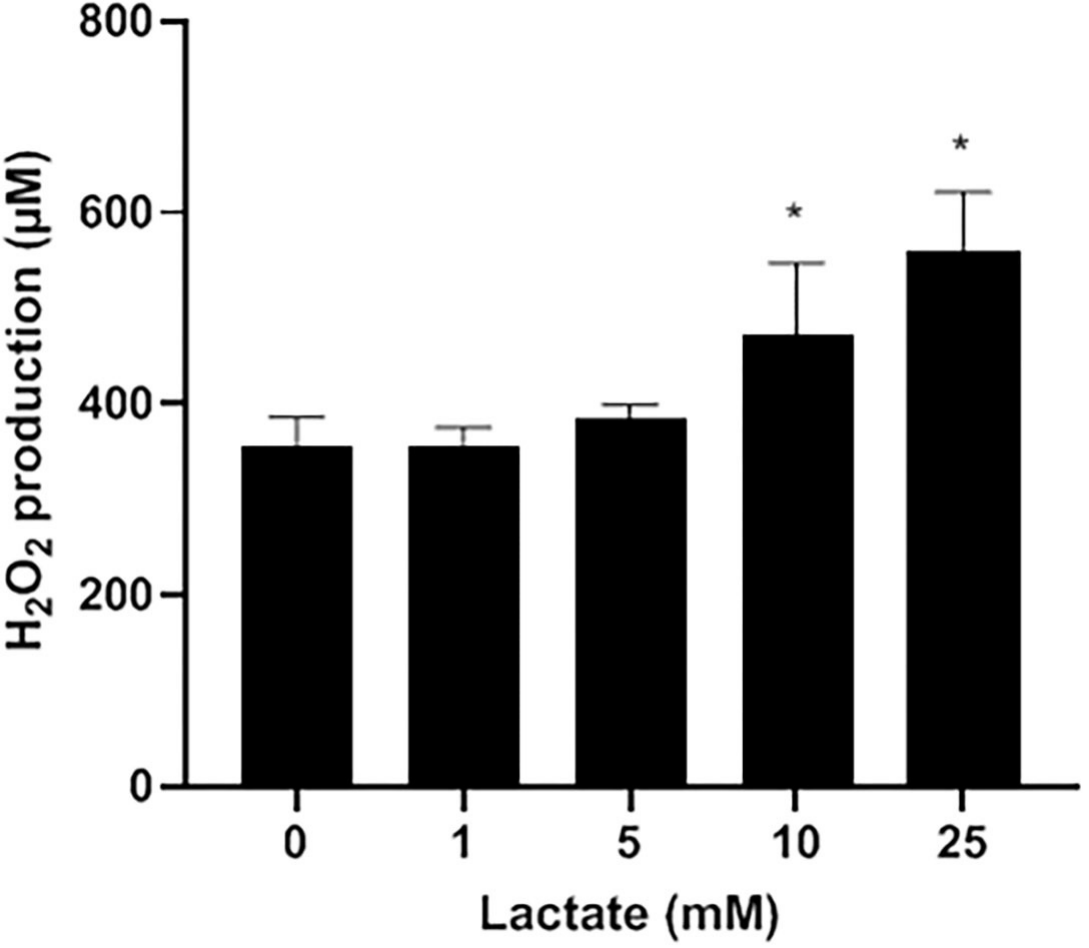
Dose-dependent effect of exogenous lactate on the production of H_2_O_2_ by *S. pluranimalium* 2N12. H_2_O_2_ was quantified using a fluorimetric assay. Assays were performed in triplicate in two independent experiments, and the means ± SD were calculated. *Significantly different at *p* < 0.01 compared to the control (no lactate).

Lactate oxidase is a bacterial enzyme that converts lactate into pyruvate and H_2_O_2_. Figure 2 indicates that exogenous lactate added to the LAPTg culture medium dose-dependently increased the amount of H_2_O_2_ produced by *S. pluranimalium*. More specifically, the presence of 25 mM lactate increased the production of H_2_O_2_ 1.57-fold compared to the control (no lactate), suggesting that the production of H_2_O_2_ by *S. pluranimalium* relies, at least in part, on the activity of lactate oxidase.

The antibacterial activity of H_2_O_2_ against *A. pleuropneumoniae* strains belonging to eighteen different serovars was evaluated by determining the MIC and MBC values. As reported in Table 1, all the strains tested had MICs and MBCs ranging from 0.57 mM (corresponding to 0.002% [v/v]) to 2.3 mM (corresponding to 0.008% [v/v]) for H_2_O_2_.

**Figure 3:**
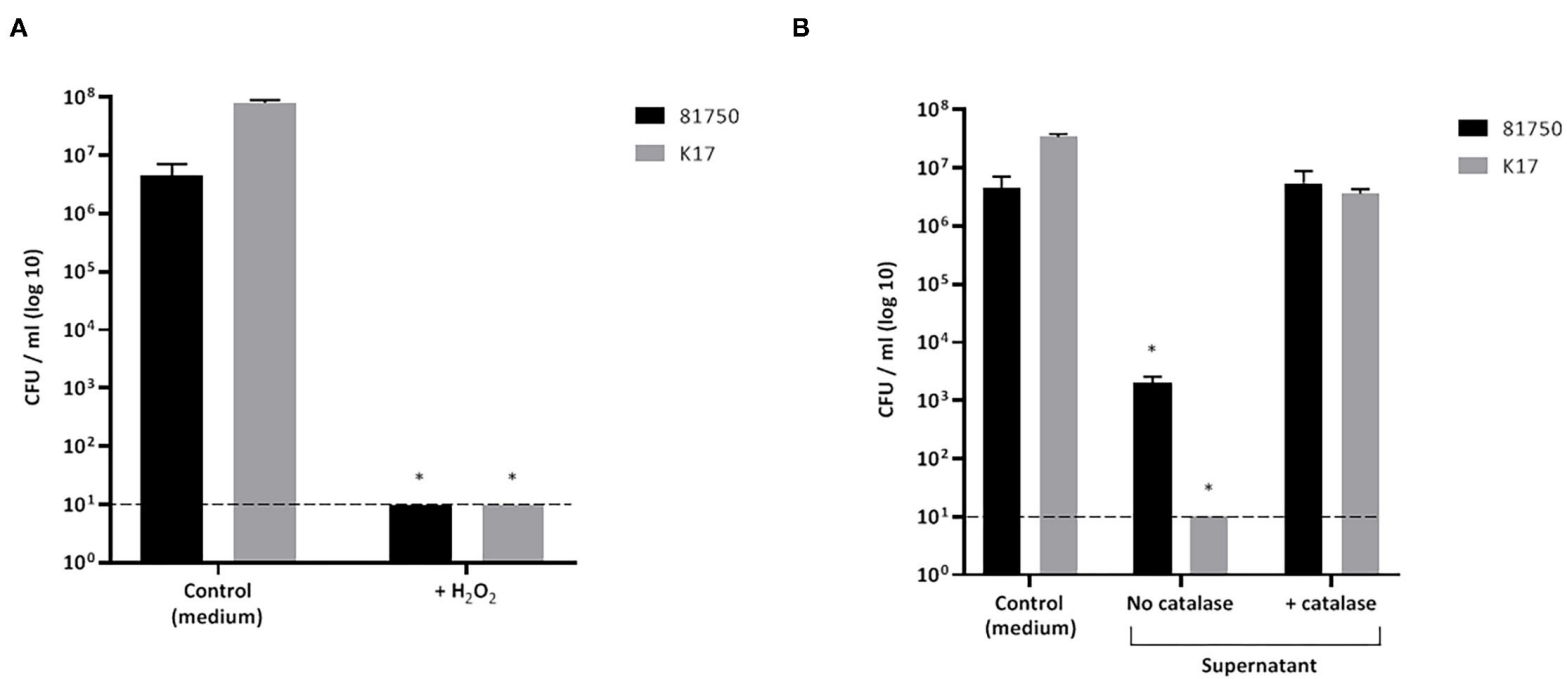
Effect of H_2_O_2_ at the minimal bactericidal concentration **(A)** and a culture supernatant of *S. pluranimalium* 2N12 treated or not with catalase **(B)** on the killing of planktonic cells of *A. pleuropneumoniae* 81750 and K17. Bacterial viability was assessed by determining colony-forming units (CFU) after an exposure time of 4 h. The broken horizontal line indicates the minimum detection limit (10^1^ CFU/mL). Assays were performed in triplicate in three independent experiments, and the means ± SD were calculated. *Significantly different at *p* < 0.01 compared to the control (no H_2_O_2_ or no *S. pluranimalium* culture supernatant).

The killing of planktonic *A. pleuropneumoniae* (strains 81750 and K17) caused by either H_2_O_2_ or a culture supernatant of *S. pluranimalium* (treated or not with catalase) after a 4-h incubation was monitored. As reported in Figure 3A, H_2_O_2_ used at a concentration of 2.3 mM, which corresponded to the MBC, reduced the viability of *A. pleuropneumoniae* 81750 (initial concentration: 5 × 10^6^ CFU/mL) and K17 (initial concentration: 4.45 × 10^6^ CFU/mL) below the minimum detection limit (10^1^ CFU/mL). On the other hand, in the control assay (no H_2_O_2_), the bacterial concentrations increased slightly. Similarly, a culture supernatant of *S. pluranimalium* (containing 0.5 mM H_2_O_2_) significantly reduced the number of planktonic *A. pleuropneumoniae* cells. Figure 3B shows that the CFUs for *A. pleuropneumoniae* 81750 dropped from 4.45 × 10^6^ CFU/mL to 2 ± 0.6 × 10^3^ CFU/mL, and from 3.4 ± 0.4 × 10^7^ CFU/mL to below the minimum detection limit (10^1^ CFU/mL) for *A. pleuropneumoniae* K17. Treating the culture supernatant of *S. pluranimalium* with catalase, which led to a residual concentration of 0.04 mM H_2_O_2_, completely abolished its bactericidal activity.

**Figure 4:**
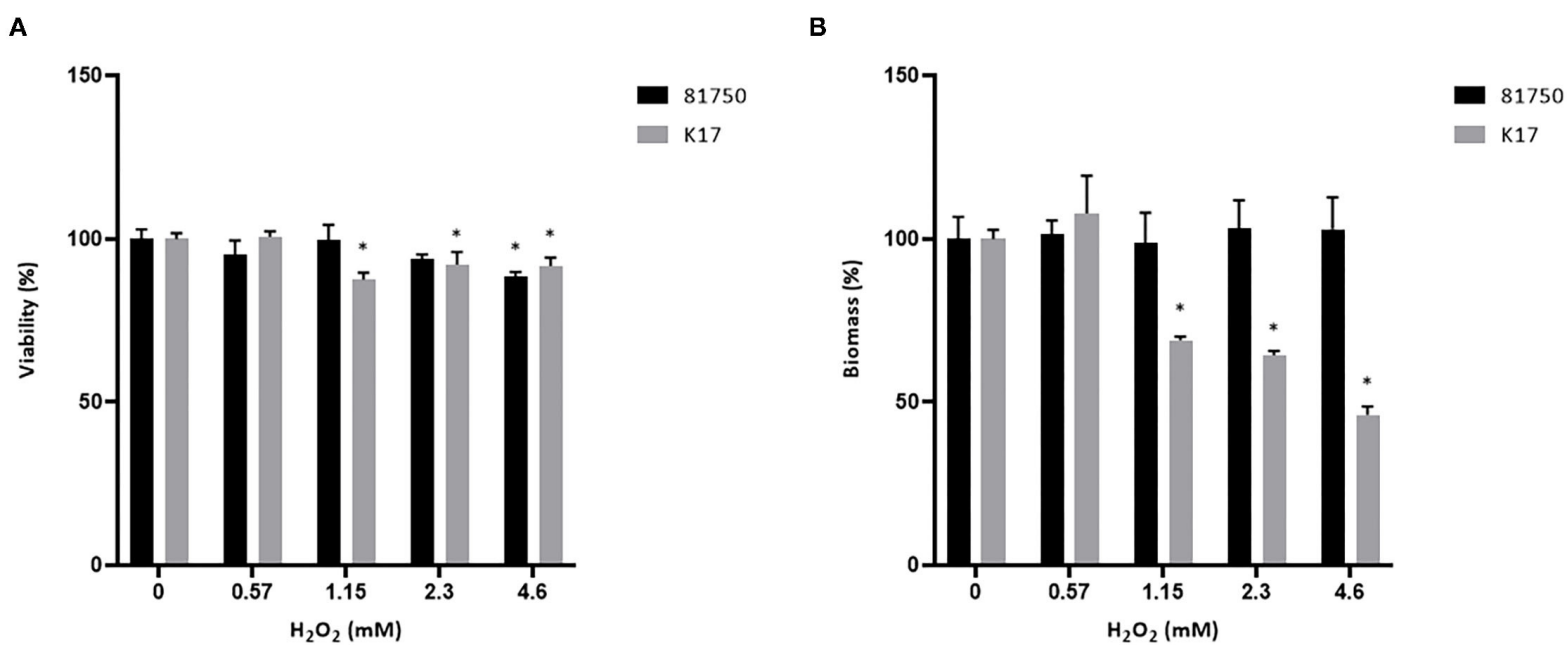
Dose-dependent effect of H_2_O_2_ on the viability **(A)** and biomass **(B)** of *A. pleuropneumoniae* 81750 and K17 biofilm-like structures following an exposure time of 4 h. Viability was assessed using the FilmTracer LIVE/DEAD Biofilm Viability kit. Biofilm-like structure biomass was determined by crystal violet staining. Assays were performed in triplicate in three independent experiments, and the means ± SD were calculated. *Significantly different at *p* < 0.01 compared to the control (no H_2_O_2_).

**Figure 5:**
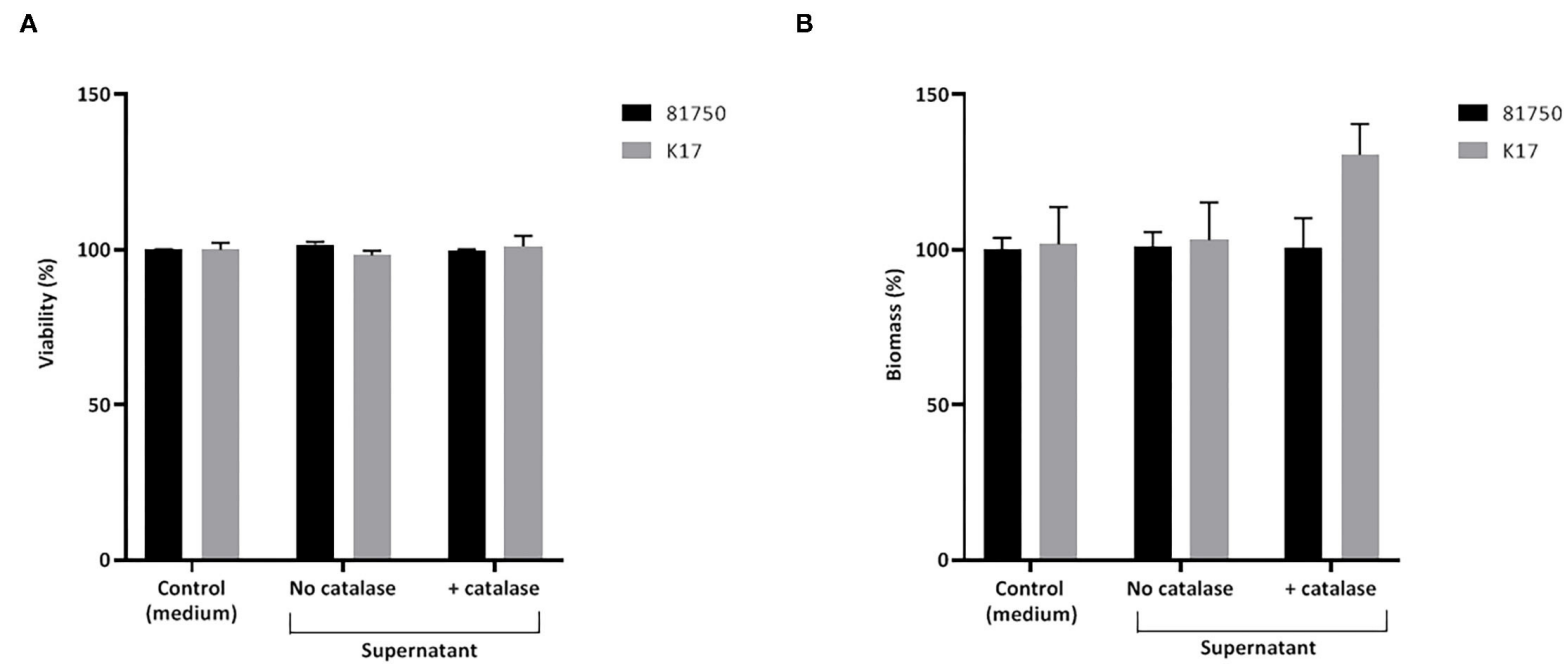
Effect of a culture supernatant of *S. pluranimalium* 2N12, treated or not with catalase, on the viability **(A)** and biomass **(B)** of *A. pleuropneumoniae* 81750 and K17 biofilms. Biofilm viability was assessed using the FilmTracer LIVE/DEAD Biofilm Viability kit. Biofilm biomass was determined by crystal violet staining. Assays were performed in triplicate in three independent experiments, and the means ± SD were calculated.

The ability of H_2_O_2_ and the culture supernatant of *S. pluranimalium* to cause the killing and desorption of biofilm-like structures of *A. pleuropneumoniae* 81750 and K17 following a 4-h treatment was then assessed. When used at a high concentration (MBC or two-fold MBC), H_2_O_2_ slightly but significantly decreased the viability of both *A. pleuropneumoniae* biofilm-like structures (Figure 4A). More specifically, at two-fold MBC (4.6 mM), viability was reduced by 11.5 ± 1.4% and 8.3 ± 2.7% for strains 81750 and K17, respectively. While H_2_O_2_ did not induce biofilm-like structure desorption for *A. pleuropneumoniae* 81750, it caused a dose-dependently desorption of the *A. pleuropneumoniae* K17 biofilm-like structure (Figure 4B). At two-fold MBC (4.6 mM), the biofilm-like biomass was reduced by 64.4 ± 1.3%. The same effects on *A. pleuropneumoniae* biofilm-like structure killing and desorption were investigated using the culture supernatant of *S. pluranimalium* containing 0.5 mM H_2_O_2_. As reported in Figure 5, no significant effects were observed.

The effect of H_2_O_2_ on the activity of conventional antibiotics, including ceftiofur, penicillin G, and tetracycline, against *A. pleuropneumoniae* 81750 and K17 was then evaluated. As reported in Table 3, synergistic effects were observed for both strains when H_2_O_2_ was used in combination with ceftiofur. Synergistic interactions were only observed for strain K17 with the H_2_O_2_/penicillin G and H_2_O_2_/tetracycline combinations.

**Table 3:** FICI values for H_2_O_2_ in combination with conventional antibiotics on *A. pleuropneumoniae*.

***A. pleuropneumoniae*** **strain**	**Antibiotic**	**FICI value^a^**	**Effect**
K17	Ceftiofur	Assay 1: 0.266	Synergy
		Assay 2: 0.133	Synergy
	Penicillin G	Assay 1: 0.5	Synergy
		Assay 2: 0.066	Synergy
	Tetracycline	Assay 1: 0.375	Synergy
		Assay 2: 0.25	Synergy
81750	Ceftiofur	Assay 1: 0.313	Synergy
		Assay 2: 0.188	Synergy
	Penicillin G	Assay 1: 1.0	No effect
		Assay 2: 0.75	No effect
	Tetracycline	Assay 1: 2.0	No effect
		Assay 2: 0.625	No effect

a *FICI ≤ 0.5 = synergistic effect, FICI > 0.5 and ≤ 1.0 = additive effect, FICI > 1.0 and ≤ 4.0 = no effect, FICI > 4.0 = antagonistic effect*.

**Figure 6:**
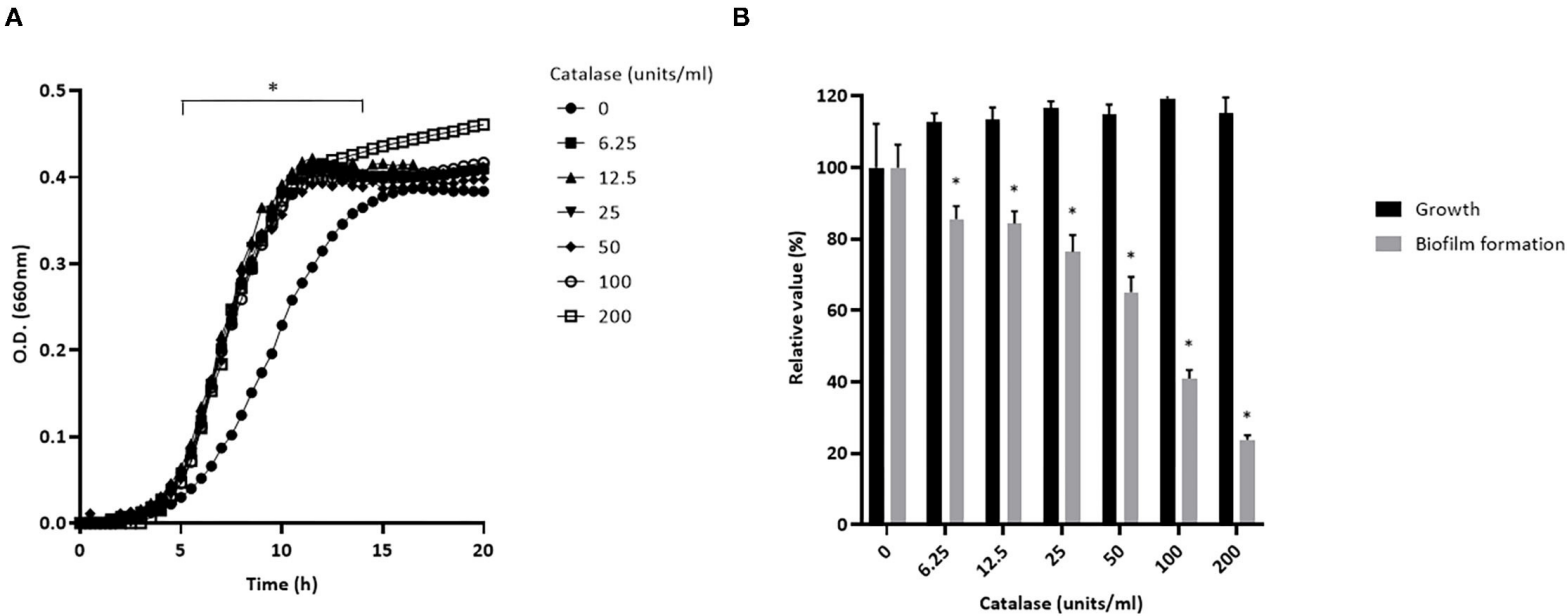
Dose-dependent effect of catalase on the growth **(A)** and biofilm formation **(B)** of *S. pluranimalium* 2N12. Bacterial growth was monitored by recording the optical density at 660 nm (OD_660_). Biofilm biomass was quantified by crystal violet staining. Assays were performed in triplicate in three independent experiments, and the means ± SD were calculated. *Significantly different at *p* < 0.01 compared to the control (no catalase).

Preliminary assays showed that *S. pluranimalium* has the ability to form a biofilm. We thus investigated whether H_2_O_2_ contributes to biofilm formation by growing *S. pluranimalium* in the presence of various amounts of catalase. On the one hand, the presence of catalase increased the bacterial growth rate, and the highest concentration tested (200 units/mL) also appeared to increase the final biomass as shown by the higher final OD_660_ (Figure 6A). On the other hand, the presence of catalase dose-dependently reduced biofilm formation (Figure 6B). More specifically, when added at a concentration of 200 units/mL, catalase decreased the formation of a biofilm by 76.3 ± 1.3%.

## Discussion

A large number of different microorganisms reside in the upper respiratory tract of pigs (11). In the healthy state, these microbial communities live in homeostasis and it can be hypothesized that they protect the animals against external pathogens that may reach this site. This protective effect may rely on the ability of certain commensal bacteria to produce antimicrobial compounds such as bacteriocins, organic acids, and hydrogen peroxide that are active against pathogenic microorganisms (12, 13). In the present study, we investigated the antagonistic activity of *S. pluranimalium* 2N12 against *A. pleuropneumoniae*.

We first confirmed an antimicrobial activity of *S. pluranimalium* 2N12 against *A. pleuropneumoniae*, which was related to H_2_O_2_ production. This is supported by the fact that the ability of a culture supernatant of *S. pluranimalium* 2N12 to induce the killing of planktonic cells of *A. pleuropneumoniae* was totally eliminated following a treatment with catalase. Moreover, commercial H_2_O_2_ was highly bactericidal for all serovars of *A. pleuropneumoniae* tested, with MBC values ranging from 0.57 to 2.3 mM. The deleterious effects of H_2_O_2_ on *A. pleuropneumoniae* may be linked to the generation of hydroxyl radicals in the presence of Fe(II) once it enters the cells (16), which results in the oxidation of macromolecules such as DNA and proteins.

Lactate oxidase (LctO) is a common H_2_O_2_-producing enzyme in bacteria that catalyzes the formation of pyruvate and H_2_O_2_ from lactate and oxygen (17). In the present study, the addition of exogenous lactate increased the production of H_2_O_2_ by *S. pluranimalium*, providing support for the key role of LctO. However, other pathways such as those involving pyruvate oxidase and amino acid oxidase (17), which may also contribute to generating H_2_O_2_, should not be excluded. Studies are currently in progress in our laboratory to investigate how environmental parameters modulate H_2_O_2_ production by *S. pluranimalium*.

As H_2_O_2_ is a harmful byproduct of aerobic metabolism and as *S. pluranimalium* does not produce catalase, it must possess a molecular mechanism to neutralize H_2_O_2_. Similarly to *S. pluranimalium, Streptococcus pneumoniae* produces large amounts of H_2_O_2_ as a byproduct of its metabolism (18). Several mechanisms have been suggested to explain how *S. pneumoniae* defends against the H_2_O_2_-mediated oxidative stress, including (i) scarcity of proteins with iron-sulfur clusters which can be damaged by reactive oxygen species (ROS) in an iron-dependent manner, (ii) expression of ferritin-like proteins known as Dps (DNA-binding protein from starved cells) or Dpr (Dps-like peroxide resistance), and (iii) production of thiol peroxidase (TpxD) activity (18). The exact mechanism that allows protection of *S. pluranimalium* from H_2_O_2_ needs to be characterized.

Evidence was brought that H_2_O_2_ production by *S. pluranimalium* is involved in its ability to form a biofilm given that the presence of catalase dose-dependently prevented biofilm formation. H_2_O_2_-mediated biofilm formation has been previously reported for *Streptococcus sanguinis* and *Streptococcus gordonii*, which are primary colonizers of human dental biofilms (19, 20). It has been proposed that H_2_O_2_ induces the release of extracellular bacterial DNA, without autolysis, that promotes cell-to-cell adhesion and biofilm formation (20).

*A. pleuropneumoniae* has the ability to form a biofilm (21, 22) that enhances its resistance to antibiotics compared to planktonic cells (23). We showed that treating a pre-formed biofilm-like structure of *A. pleuropneumoniae* with H_2_O_2_ at a concentration corresponding to the MBC (2.3 mM) reduces its viability. No killing of the biofilm-like structure was obtained with a culture supernatant of *S. pluranimalium*, which is likely related to the fact that the supernatant contained a low amount of H_2_O_2_ (0.5 mM). Additional studies are required to demonstrate the killing effect of H_2_O_2_ using more relevant biofilm models of *A. pleuropneumoniae*.

The effect of H_2_O_2_ on the activity of conventional antibiotics used to treat *A. pleuropneumoniae* infections was assessed using the checkerboard technique. Synergistic interactions between H_2_O_2_ and some antibiotics, including ceftiofur, penicillin G, and tetracycline, were demonstrated, particularly against *A. pleuropneumoniae* K17. This observation was in agreement with the study of Sgibnev and Kremleva (24) who investigated the influence of various microbial metabolites on the antibiotic sensitivity of bacteria. The authors reported that H_2_O_2_ was the most effective bacterial metabolite for increasing the sensitivity of both Gram-positive and Gram-negative bacteria to several antibiotics. They proposed that H_2_O_2_ may cause a shift in the balance of pro-oxidants and antioxidants in bacteria and that the resulting oxidative stress enhances the effects of antibiotics on the target bacteria.

Based on our results, the production of H_2_O_2_ by *S. pluranimalium* could be regarded as a potential protection mechanism of the upper respiratory tract against H_2_O_2_-sensitive pathogens such as *A. pleuropneumoniae*. Interestingly, previous studies have documented the role of H_2_O_2_ produced by commensal streptococci colonizing the oral cavity in controlling cariogenic bacteria (mainly *Streptococcus mutans*) (25–27). It is worth mentioning that the presence of catalase positive staphylococci in the upper respiratory tract of pigs (11) may attenuate the beneficial impact of H_2_O_2_.

The resistance of *A. pleuropneumoniae* to various antibiotics, including tetracycline, ampicillin, and penicillin, is on the increase and is a growing concern (28–30). The identification of alternative strategies for controlling *A pleuropneumoniae* infections is thus of great interest. In this regard, further studies are required to explore the possibility of using *S. pluranimalium* as a probiotic to antagonize respiratory pathogens such as *A. pleuropneumoniae*.

## Conclusions

Based on our results, the production of H_2_O_2_ by *S. pluranimalium* could be regarded as a potential protective mechanism of the upper respiratory tract against H_2_O_2_-sensitive pathogens such as *A. pleuropneumoniae*.

## Data Availability Statement

The original contributions presented in the study are included in the article/supplementary material, further inquiries can be directed to the corresponding author.

## Author Contributions

MF, MG, and DG conceived and designed the experiments. KV performed the experimental assays and the statistical analysis. DG drafted and finalized the manuscript. All authors contributed to the article and approved the submitted version.

## Funding

This study was supported by Fonds de recherche du Québec—Nature et technologies (2018-PR-205462).

## Conflict of Interest

The authors declare that the research was conducted in the absence of any commercial or financial relationships that could be construed as a potential conflict of interest.

## Publisher's Note

All claims expressed in this article are solely those of the authors and do not necessarily represent those of their affiliated organizations, or those of the publisher, the editors and the reviewers. Any product that may be evaluated in this article, or claim that may be made by its manufacturer, is not guaranteed or endorsed by the publisher.
